# Sex-specific Estrogen Levels and Reference Intervals from Infancy to Late Adulthood Determined by LC-MS/MS

**DOI:** 10.1210/clinem/dgz196

**Published:** 2019-11-13

**Authors:** Hanne Frederiksen, Trine Holm Johannsen, Stine Ehlern Andersen, Jakob Albrethsen, Selma Kløve Landersoe, Jørgen Holm Petersen, Anders Nyboe Andersen, Esben Thyssen Vestergaard, Mia Elbek Schorring, Allan Linneberg, Katharina M Main, Anna-Maria Andersson, Anders Juul

**Affiliations:** 1 Department of Growth and Reproduction, Rigshospitalet, University of Copenhagen, Copenhagen, Denmark; 2 International Center for Research and Research Training in Endocrine Disruption of Male Reproduction and Child Health (EDMaRC), University of Copenhagen, Copenhagen, Denmark; 3 The Fertility Clinic, Rigshospitalet, University of Copenhagen, Copenhagen, Denmark; 4 Department of Biostatistics, University of Copenhagen, Copenhagen, Denmark; 5 Paediatrics and Adolescent Medicine, Aarhus University Hospital, Aahus, Denmark; 6 Center for Clinical Research and Disease Prevention, Bispebjerg and Frederiksberg Hospital, University of Copenhagen, Copenhagen, Denmark; 7 Department of Clinical Medicine, Faculty of Health and Medical Sciences, University of Copenhagen, Copenhagen, Denmark

**Keywords:** estradiol, estrone, reference range, LC-MS/MS, mini-puberty, childhood, pubertal development, menstrual cycle, menopause

## Abstract

**Context:**

The lack of sensitive and robust analytical methods has hindered the reliable quantification of estrogen metabolites in subjects with low concentrations.

**Objective:**

To establish sex-specific reference ranges for estrone (E1) and estradiol (E2) throughout life and to evaluate sex-differences using the state-of-the-art liquid chromatography tandem mass spectrometry (LC-MS/MS) method for quantification of E1, E2, and estriol (E3).

**Design:**

LC-MS/MS method development and construction of estrogen reference ranges.

**Settings:**

Population-based cross-sectional cohorts from the greater Copenhagen and Aarhus areas.

**Participants:**

Healthy participants aged 3 months to 61 years (n = 1838).

**Results:**

An isotope diluted LC-MS/MS method was developed and validated for measurements of serum E1, E2, and E3. Limits of detections (LODs) were 3 pmol/L (E1), 4 pmol/L (E2), and 12 pmol/L (E3), respectively. This sensitive method made it possible to differentiate between male and female concentration levels of E1 and E2 in children. In girls, E2 levels ranged from <LOD to 100 pmol/L during mini-puberty, whereas it was ≤20 pmol/L during childhood. E1 and E2 increased with age and pubertal breast stage and varied during the menstrual cycle; E1 was lower than E2 in girls and premenopausal women, and higher than E2 in postmenopausal women. In boys, E1 and E2 increased with age and pubertal stage, whereas little changes with age were observed in men. High E3 concentrations were confirmed in pregnant women.

**Conclusion:**

Reference ranges of simultaneous quantification of E1 and E2 by this novel specific and highly sensitive LC-MS/MS method provide an invaluable tool in clinical practice and in future research studies.

Concentrations of circulating estrogens show a wide variation in healthy subjects throughout life. The diagnostic use of estradiol (E2) measurement includes the evaluation of girls with premature thelarche, precocious and delayed puberty, boys and men with gynecomastia, patients with suspected hypogonadism, as well as monitoring of hypogonadal women during hormone replacement, and women with breast cancer during hormone suppressive therapy. In most clinical situations of girls and premenopausal women E2 measurement may be sufficient, whereas in postmenopausal women estrone (E1) is the most prevalent estrogen metabolite and may therefore be more relevant to assess. Furthermore, as part of clinical and diagnostic evaluation of men with gynecomastia, E1 and estrone sulfate (E1-S) levels might be equally relevant compared with those of E2. High accuracy, specificity, and precision as well as standardization of E2 assays are considered mandatory nowadays, according to the Endocrine Society (1–3). Sensitive mass spectrometry-based methods, such as the liquid chromatography tandem mass spectrometry (LC-MS/MS) methods, are now accepted as state-of-the-art methods for quantitative analysis of E2 and many other steroid hormones in serum (4).

Nevertheless, most currently used methods do not reliably quantify estrogen metabolites, especially in the very low range. Measurement by immunoassays, which are susceptible to cross-reactivity, are still in widespread use and the majority of previously reported E2 concentrations determined by different immunoassays are most likely incorrect and vary markedly from assay to assay, especially in the low range (5). With the use of larger sample volume (500 µL), highly sensitive radioimmunoassays (RIAs) have been reported to be able to quantify E2 in the low concentration range (<40 pmol/L or even lower) (6). However, larger sample volumes can be difficult to obtain from infants and small children and do not address the issue of potential cross-reactivity. Sensitive and specific E2 assays are not needed in ovulatory women of fertile age but could be essential in the evaluation of individuals with low E2 concentrations such as in infants, prepubertal children of both sexes, adolescent boys, and men, and such assays may serve a purpose for postmenopausal women and in hypogonadotropic amenorrhoeic patients. In these conditions, sensitive and specific assays as well as detailed references ranges for estrogen metabolites are needed.

Several gas chromatography (GC) MS or LC-MS/MS methods have been developed during recent decades, but few methods meet all of the requirements for clinical purposes, including high accuracy, precision, and sensitivity; a realistic sample volume; and a short runtime with easy pre- and postsample preparation. To achieve a sufficiently high sensitivity, many GC-MS and LC-MS/MS methods use chemical derivatization to improve the stability and ionization efficiency of E2 (7–9). This involves a relatively time- and manpower-consuming process, and some researchers have recently succeeded in developing methods in which the derivatization steps have been avoided. Instead, steroids including E2 and E1 have been extracted from the serum by simple liquid–liquid extraction procedures. In addition, ionization has been improved by using an ammonium fluoride buffer instead of an ammonium acetate buffer as mobile phase (10–15). Meanwhile, LC-MS/MS instruments have in general become more sensitive, making LC-MS/MS a suitable technology for sensitive steroid measurements.

Here we present a new isotope-diluted online TurboFlow LC-MS/MS method with liquid–liquid extraction. The method is validated for the entire range of concentrations of estrogens throughout life. Detailed sex-specific reference ranges for estrogens from large cohorts of healthy children and adults are presented, and the impact of sex, age, pubertal stage, menstrual cycle day, use of oral contraceptives, and menopausal status on estrogens concentrations is evaluated.

## Material and Methods

### Subjects

In total, serum samples from 1838 healthy subjects (772 boys and men/1066 girls and women) selected from 5 different cohorts were analyzed for estrogen concentrations: cohort 1, a subset from the Copenhagen Mother–Child Cohort: 50 boys and 50 girls aged 3.0 to 5.3 months, 17 boys and 15 girls aged 10 to 14 months, and 49 boys and 48 girls aged 4.2 to 6.0 years (http://www.edmarc.net/mother-child-cohort.html, (16,17); cohort 2, a subset of 48 girls aged 7.9 months to 6.0 years from the study of the follicle-stimulating hormone (FSH) and luteinizing hormone (LH) response to a gonadotropin-releasing hormone test in this age group (18); cohort 3, 499 boys and 604 girls aged 5.9 to 23.4 years and additionally 62 adolescent girls using oral contraceptives aged 14.8 to 19.4 years from the Copenhagen Puberty Study (http://www.edmarc.net/puberty-cohort.html, (19,20); cohort 4, 157 men aged 31.4 to 60.9 years and 93 postmenopausal women aged 54.6 to 60.7 years from Health2008, a population-based cross-sectional study conducted at the Research Centre for Prevention and Health, Glostrup University Hospital, Denmark (21,22); and cohort 5, 194 women aged 24.7 to 43.9 years with regular menstrual cycles. The women were seen in the Fertility Assessment and Counselling Clinic (FAC Clinic), Copenhagen University Hospital between 2012 and 2016 (23).

### Clinical examinations

In all cohorts, participants were examined for their general health status according to the respective study protocols. In cohort 3, pubertal stage was assessed by clinical examination, breast stages (B1–B5) by palpation, and genital stage (G1–G6) by inspection according to the classification by Marshall and Tanner (19,20,24,25), and information on age at menarche and on use of oral contraceptives were given as self-reported data in a questionnaire (19). In cohort 5, information on the given menstrual cycle day (the day the blood samples were withdrawn) was obtained by the examining physician (23).

### Hormone analysis

All blood samples were drawn from an antecubital vein, clotted and centrifugated, and serum was stored at –20°C until analysis. All hormone analyses were performed at Department of Growth and Reproduction, Rigshospitalet, Copenhagen, Denmark. For analysis of E1 and E2, a new method for simultaneous quantitative determination of estrogens, also including estriol (E3) in human serum by isotope dilution online TurboFlow-LC-MS/MS was developed and validated (all supplementary material and figures are located in a digital research materials repository (26)).

In short, after the thawing of 200 µL of each serum sample, isotope-labelled internal standards were added to serum calibration and control samples, and the estrogens were purified from the serum by liquid–liquid extraction using heptane/ethyl acetate. The analysis was performed on a Dionex UltiMate 3000 UHPLC system with integrated Transcend TLX TurboFlow sample preparation system coupled with triple quadrupole mass spectrometer (TSQ Quantiva) from Thermo Scientific controlled by Aria MX 2.2 and Xcalibur 4.0 software. For further sample extraction and chromatographic separation of the estrogens, the TurboFlow-LC system was equipped with a loading Cyclone-P TurboFlow column followed by an analytical Kinetex® Phenyl-Hexyl column. The MS/MS system was equipped with a heated electrospray ionization source (HESI) running in negative mode. The total duration time was 5.50 minutes. Injection volume, flow rate, solvents and solvent programming, optimized MS/MS interphase settings and MS transitions, retention times, collision energies, and S-lens settings optimized for each single analyte are shown in the supplementary material see (26). LC-MS/MS-extracted ion chromatograms of E1, E2, and E3 including internal standards, qualifier, and quantifier ions in the lowest calibration standard are shown in (26).

By linear regression based on area ratios (sample area/internal standard area) the concentrations of unknown samples and control material were determined. For method validation and all other analyses two calibration curves in Milli-Q water were included at the beginning and the end of all sample batches.

Method validation was based on repeated calibration curves made in a serum pool and Milli-Q water. Matrix effect, ion suppression, linearity, limit of detection (LOD), and quantification (LOQ) were calculated, and the intraday variability including accuracy (% recovery) and precision (relative standard deviation (RSD)) for a low, medium, and high concentration levels (Q low, Q middle, and Q high) were estimated using the regression function in Analysis Toolpak for Microsoft Excel 2007 according to international guidelines (27). The interday variation (precision) was estimated from the analysis of control material analyzed in duplicates in 20 different batches during a period of 2 months. Validation of the new estrogen LC-MS/MS method for clinical use included testing of stability in full blood and serum, storage at different temperatures over time, repeated freezing/thawing cycles, and interference by hemolysis. Our lab participates in the external quality assessment program *Steroid Hormones* from the United Kingdom National External Quality Assessment Service (28). The analysis is accredited by the Danish Accreditation Fund (29) according to the DS/EN 15189 standard for medical laboratories. All serum samples used in the present study for establishment of sex- and age-specific reference ranges were analyzed in 48 batches over a period of 8 months. Each batch included standards for calibration curves, approximately 40 unknown samples, 2 blanks, and a total of 8 control samples, including 2 serum pool controls, and 2 sets of serum pool controls spiked with native estrogen standards at low, medium, and high levels, and an unspiked serum pool.

Progesterone, 17-hydroxyprogesterone (17-OHP), androstenedione, testosterone (T), dehydroepiandrosterone sulfate (DHEAS), and E1S were analyzed in cohort 4 samples by LC-MS/MS as previously described (18). Quantification limits were 0.036 nmol/L (progesterone), 0.1 nmol/L (17-OHP), 0.042 nmol/L (androstenedione), 0.012 nmol/L (T), 19 nmol/L (DHEAS), and 0.026 nmol/L (E1-S). Samples were analyzed in 4 batches. The interday variation expressed as the relative standard deviation (RSD) were <6% for these 6 steroids.

Until September 2014, serum concentrations of sex hormone-binding globulin (SHBG) were determined by a time-resolved fluoroimmunoassay (AutoDELFIA; PerkinElmer, Turku, Finland) with a LOD of 0.23 nmol/L and interassay coefficients of variation (CVs) of the SHBG assay of <7%. The assay has previously been validated in our laboratory (30). From September 2014 onward, SHBG was determined by a chemiluminescence immunoassay (Access2, Beckman Coulter, Brea, CA, USA) with a LOD of 0.33 nmol/L and interassay CV of the SHBG assay of <9%.

### Calculations and statistics

Free estradiol (free E2) and free estrone (free E1) were calculated based on Mazer (31), who determined new constants, mean K_SHBG_x_-values, and mean of K_Alb_x_-values based on SHBG and albumin data from 6 different studies and integrated them into the original equation by Vermeulen et al. (32):

$$\begin{array}{l} X=\frac{X_{total}}{\left(1+K_{SHBG_{X}}*\frac{\left[SHBG\right]}{MW_{SHBG}}+K_{Alb_{X}}*\frac{\left[Alb\right]}{MW_{Alb}}+K_{CBG_{X}}*\frac{\left[CBG\right]}{MW_{CBG}}\right)} \end{array}$$

where X = E1 or E2

In our calculations, binding to cortisol binding globulin (CBG) was not included due to missing cortisol concentrations. Thus, the equation for free E2 (or E1) is:

$$\begin{array}{l} Free\;E2=\frac{E2measured\;pmol/L\;}{1+\left(\frac{K\;SHBG_{E2}\frac{L}{mol}*SHBG\;measured\frac{g}{L}}{MW\;SHBG\frac{g}{mol}}\right)+\left(\frac{KALB_{E2}\frac{L}{mol}*43\frac{g}{L}}{MW\;ALB\frac{g}{mol}}\right)}=\;XX\;pmol/L\; \end{array}$$

where the binding constants used were 5 × 10^8^ L/mol (K SHBG_E2_), 4.55 × 10^4^ L/mol (K ALB_E2_), 9.4 × 10^7^ L/mol (K SHBG_E1_), and 3.6 × 10^4^ L/mol (K SHBG_E1_) according to Mazer (31).

Reference charts were developed using the Generalized Additive Models for Location, Scale and Shape (GAMLSS). In the analysis, the data were summarized in 3 smoothed age-dependent curves: L, M, and S, of which the L-curve adjusts age-dependent skewness, the M curve corresponds to the age-dependent median, and the S-curve is approximately the age-dependent coefficient of variation. The applied method was based on the Box–Cox power transformation, which transformed the data to follow a Gaussian distribution for each age. Calculation of the standards deviation (SD) score was based on the following equation:

$$\begin{array}{l} SD\;Score=\frac{{\left(\frac{X}{M}\right)}^{L}-1}{L\;\times \;S}. \end{array}$$

where X was the hormone concentration and L ≠ 0 (33).

The GAMLSS method allows back-calculation to obtain selected percentiles in the original distribution. In the reference charts, we show the 2.5th, 16th, 50th, 84th, and 97.5th percentiles corresponding to mean – 2 × SD, mean – 1 × SD, mean, mean + 1 × *SD, and mean + 2 × SD, respectively. GAMLSS analysis was performed using R (version 3.5.2; R Core Team, R Foundation for Statistical Computing, Vienna, Austria (http://www.R-project.org/)) and the GAMLSS package.

For graphic illustration of estrogen reference areas, the concentration values of E1 and E2 below LODs, that is, 2.93 pmol/L and 4.04 pmol/L, respectively, were replaced with values of 1 pmol/L. The corresponding calculated values of free E1 and free E2, where E1 and E2 concentrations were below LODs, were replaced with the value 0.01 pmol/L.

To compare medians across menstrual cycle groups (categorized as day 1–7, 8–14, and 15+), the 2-tailed Mann–Whitney U-test was used. *P* < .05 was considered statistically significant. Calculations were performed using IBM SPSS Statistics, version 22 (IBM Corporation, Armonk, NY, USA). Graphical presentations were performed in Excel and GraphPad Prism 7.

### Ethical considerations

For all participants, a written, informed consent form was signed before study participation, either by the participant or by their parents, in the case of children below 18 years. The cohort studies were approved by the ethics committees of the Capital Region of Denmark (RegionH): cohort 1, no. [KF] 01-030/97; cohort 2, no. 1-10-72-631-12, the study is registered in ClinicalTrials.gov (identifier NCT01944488); cohort 3, no. KF 01 282214 and V200.1996/90, the study is registered in ClinicalTrials.gov (identifier NCT01411527); cohort 4, no. H-KA20060011; and cohort 5, no. H-I-2011–081. Furthermore, the studies were approved by the Danish Data Protection Agency: cohort 1, no. 2003-41-2996; cohort 2, no. 2007-58-0010; cohort 3, no. 2010-41-5042; cohort 4, RH-2015–299; and cohort 5, no. 2012-58-0015. The present study is in accordance with the Helsinki II declaration.

## Results

### Estrogen LC-MS/MS method validation

**Figure 1.**
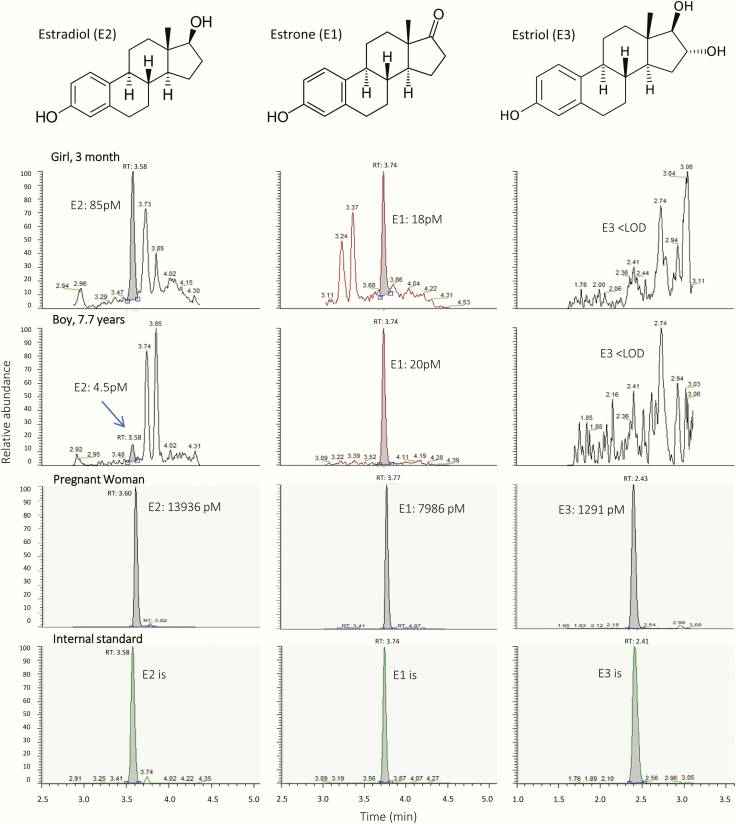
Chemical structures of the estrogens and LC-MS/MS extracted ion chromatograms of estrone (E1), estradiol (E2), and estriol (E3) in serum from a 3-month-old girl, a prepubertal boy, and a pregnant woman.

All calibration curves were linear in the measuring range 3.7 to 2655 (E1), 3.6 to 2570 (E2) and 20.8 to 7282 pmol/L (E3) with correlation coefficients (r^2^) > 0.99. LODs were 2.93 (E1), 4.04 (E2), and 12.3 pmol/L (E3). Calibration curves prepared in human serum and in Milli-Q water had identical slopes and no matrix effects or ion suppression were observed (26). The accuracies expressed as percent recovery for all 3 estrogens in all control materials (Q low, Q middle and Q high) were above 97%. The intraday precisions expressed as relative standard deviation (RSD) for Q middle and Q high were ≤7.5% and <20% for Q low, while the interday precision for Q middle and Q high were ≤8.9% and were ≤11% for Q low (26). Stability experiments showed that the 3 estrogens were stable in serum stored at 4°C and 25°C for at least 6 days. E1 and E2 were also stable in full blood (E3 was not tested). All 3 estrogens were stable in serum samples after at least 3 freezing/thawing cycles. All 3 estrogens were stable in hemolyzed serum up to a hemolysis degree of 0.56 mmol/L (data not shown). Moreover, based on 8 distributions (38 samples) from UK-NEQAS, the E2-results from our lab had a mean bias of +7.0% when compared with other labs using LC-MS/MS technology. Fig. 1 shows the chemical structures and LC-MS/MS extracted ion chromatograms of E1, E2, and E3 in serum from a 3-month-old girl, a prepubertal boy, and a pregnant woman.

### Estrogen reference ranges

In total, E2 was detectable in 96% and 63% of all female and male serum samples, respectively, while E1 was detectable in 89% in both sexes. From infancy until puberty onset E2 was detectable in 68% and 22% of the female and male samples, respectively, while E1 was detectable in 88% (female) and 74% (male). E3 was only detectable in very few samples in the concentration range between LOD (12.3 pmol/L) and LOQ (37 pmol/L).

### Girls and women. 

**Figure 2.**
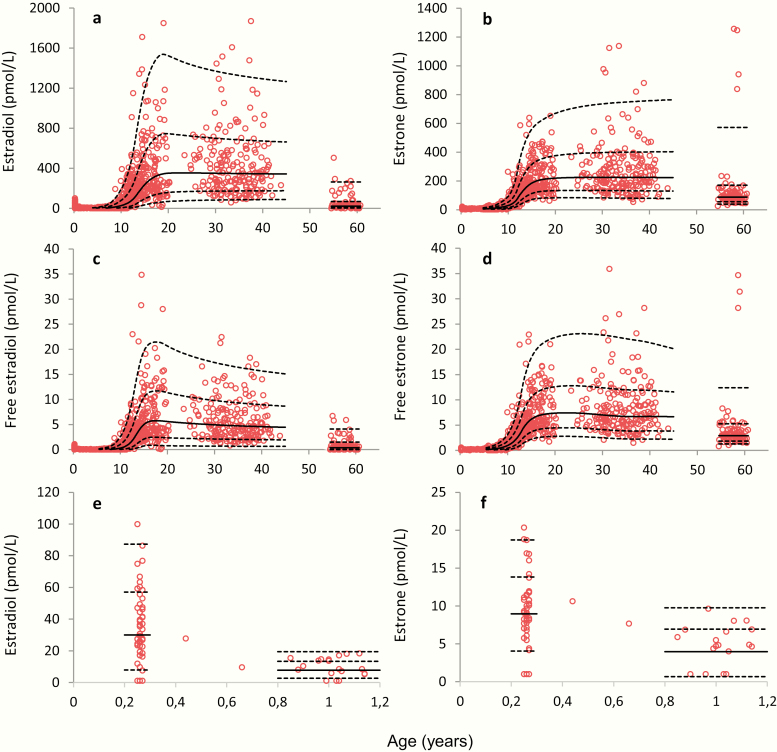
Female serum concentrations of (a) estradiol (E2) and (b) estrone (E1) as a function of age from 0.25 to 61 years, n = 1007; calculated (c) free E2 and (d) free E1 from 0.25 to 61 years, n = 936 and (e) E2 and (f) E1 from 0.25 to 1.14, years n = 65. Solid lines represent medians (50 percentile), and dashed lines represent the 2.5, 16, 84, and 97.5 percentiles (corresponding to ± 1 and ± 2 standard deviations, respectively).

Serum concentrations of E1, E2, free E1, and free E2 as a function of age are shown in Fig. 2. In infant girls, both E1 and E2 concentrations were significantly higher around 3 months of age (0.25 years) than those of older prepubertal girls (*P* < .001, Fig. 2e and 2f), and the median E2 concentration was significantly higher than median E1 concentration in girls around 3 months (*P* < .001) and 1 year of age (*P* = .001). In contrast, from around 1 to 7 years of age the median E1 concentration was significantly higher than median E2 (*P* < .001). From around 10 years of age, E1 and E2 concentrations as well as calculated free levels of E1 and E2 significantly increased with increased age and peaked around 15 to 16 years of age (Fig. 2c and 2d). The adult median E2 concentration was relatively constant up to +40 years, but with a large interindividual variation presumably reflecting differences related to pubertal timing and menstrual cycle day at blood sampling (see later). The women´s median E2 concentration was significantly higher than their median E1 concentration.

### Menopausal status.

Among women aged 55 to 61 years, E2 concentrations were significantly lower than in younger women, but detectable in more than 94%, ranging from 7 to 80 pmol/L in these presumably postmenopausal women (Fig. 2a). Twelve (13%) women above 55 years had E2 serum concentrations >100 pmol/L, and 5 women (5.4%) had very high E1 serum concentrations (≥838 pmol/L).

Fig. 2c and 2d presents the calculated free E1 and free E2 according to age and are shown for girls and women. Concentrations of E1, E2, free E1, and free E2 according to age plotted on a logarithmic scale are shown for girls and women in (26).

### Boys and men.

**Figure 3.**
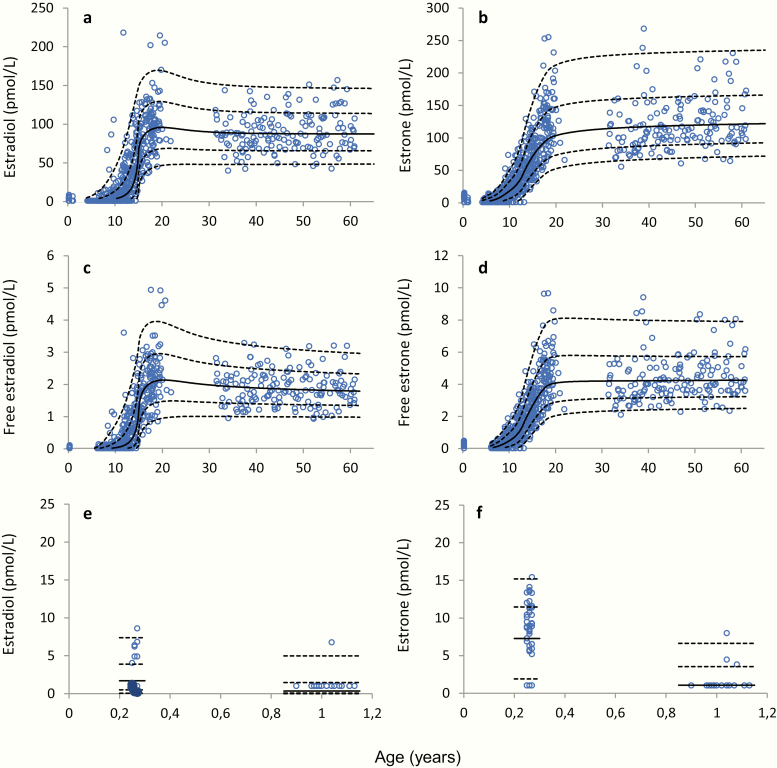
Male serum concentrations of (a) estradiol (E2) and (b) estrone (E1) as a function of age, from 0.2 to 60 years, n = 772; calculated (c) free E2 and (d) free E1 from 0.25 to 61 years, n = 706; and (e) E2 and (f) E1 from 0.2 to 1.13 years, n = 67. Solid lines represent medians (50 percentile), and dashed lines represent the 2.5, 16, 84, and 97.5 percentiles (corresponding to ±1 and ±2 standard deviations, respectively).

Serum concentrations of E2 and E1 in boys and men according to age are shown in Fig. 3. In boys and men, the median E2 concentration was in general significantly lower than the median E1 concentration (Fig. 3a and 3b), and the calculated free E1 was almost twice as high as the free E2 in men (Fig. 3c and 3d). In infancy, E2 was only detectable in concentrations above LOD in 14% of the samples, while E1 was detectable in most boys (82%) at 3 months of age (Fig. 3e and 3f). Concentrations of E1, E2, free E1, and free E2 according to age plotted on a logarithmic scale are shown in (26).

### Association to pubertal stage

#### Girls. 

**Figure 4.**
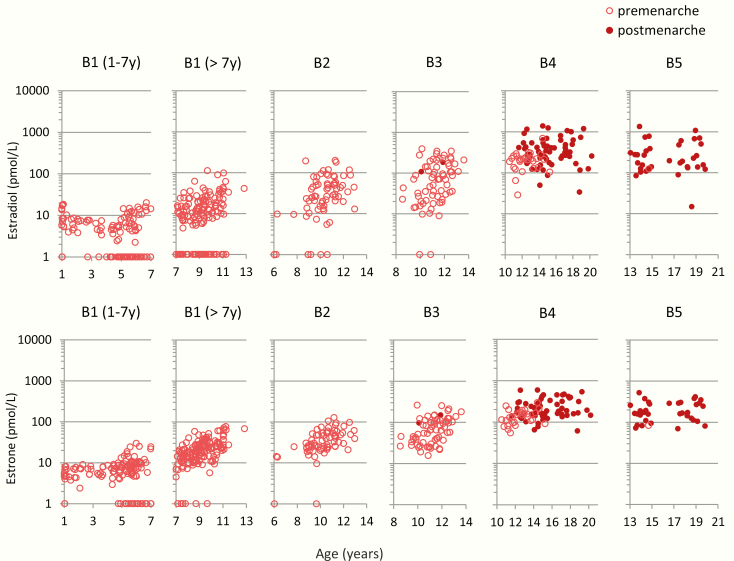
Female serum concentrations (n = 456) of estradiol (E2) and estrone (E1) by age, stratified by Tanner breast stages B1–B5: B1, n = 178; B2, n = 71; B3, n = 69; B4, n = 95; and B5, n = 43. Number of adolescents postmenarche (filled circles), n = 112.

**Figure 5.**
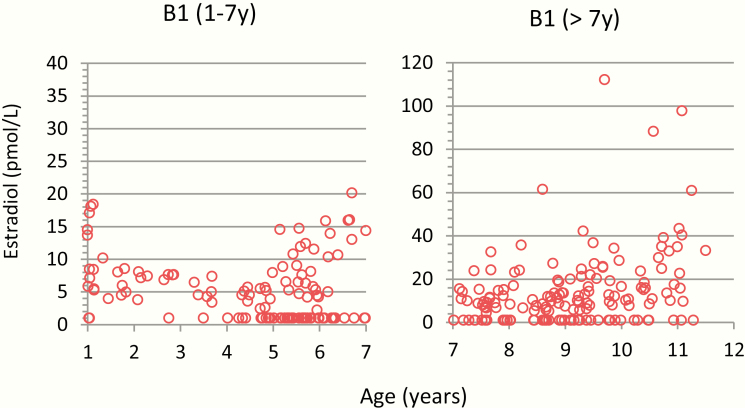
Serum concentrations of estradiol (E2) and estrone (E1) in prepubertal girls, Tanner stage B1 before and after 7 years of age, in total n = 178. Please note the different y-axes.

The serum concentrations of E1 and E2 in 545 prepubertal and pubertal girls stratified by Tanner breast stage B1–B5 are shown in Fig. 4. The highest measured E2 concentration in prepubertal girls below 7 years of age was 20 pmol/L (Fig. 5). Older prepubertal girls (>7 years) had slightly higher E2 concentrations, which increased with age (Figs. 4 and 5). Almost all E2 concentrations were above 10 pmol/L in girls with breast stage ≥B2 and above 100 pmol/L in girls with breast stages B4–B5. Furthermore, the presumed postmenarcheal women had higher serum E2 concentrations than premenarcheal girls in stages B4–B5. (Figs. 3 and 4). A similar pattern was observed for E1, in which majority of the prepubertal girls below 7 years of age had E1 concentrations <10 pmol/L (highest concentration was 30 pmol/L), and most of the prepubertal girls above 7 years of age had E1 concentrations in the range of 10 to 100 pmol/L (Fig. 4). Free E1 and free E2 concentrations for prepubertal and pubertal girls are shown in (26).

#### Boys. 

**Figure 6.**
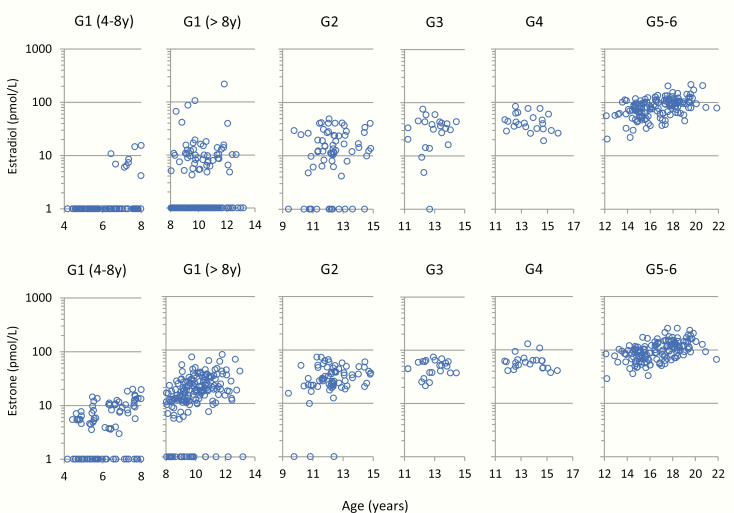
Male serum concentrations (n = 537) of estradiol (E2) and estrone (E1) by age, stratified by Tanner genital stages G1–G6. G1 (4–8 years), n = 95; G1 (>8 years), n = 180; G2, n = 69; G3, n = 23; G4, n = 23; and G5–6, n = 146.

Both E1 and E2 increased with increasing age and pubertal stage. In most boys, both estrogens were >10 pmol/L at G2 or above. In prepubertal boys, we observed different estrogen levels for boys below and above 8 years of age, respectively (Fig. 6). In the youngest prepubertal boys (4–8 years), both E1 and E2 concentrations were <20 pmol/L in all individuals, while among the older (>8 years) prepubertal boys, especially E1 in the older (>8 years) prepubertal boys was in the range of 10 to 100 pmol/L (Fig. 6). Free E2 and free E1 concentrations for prepubertal and pubertal boys are shown in (26).

### Association to menstrual cycle

**Figure 7.**
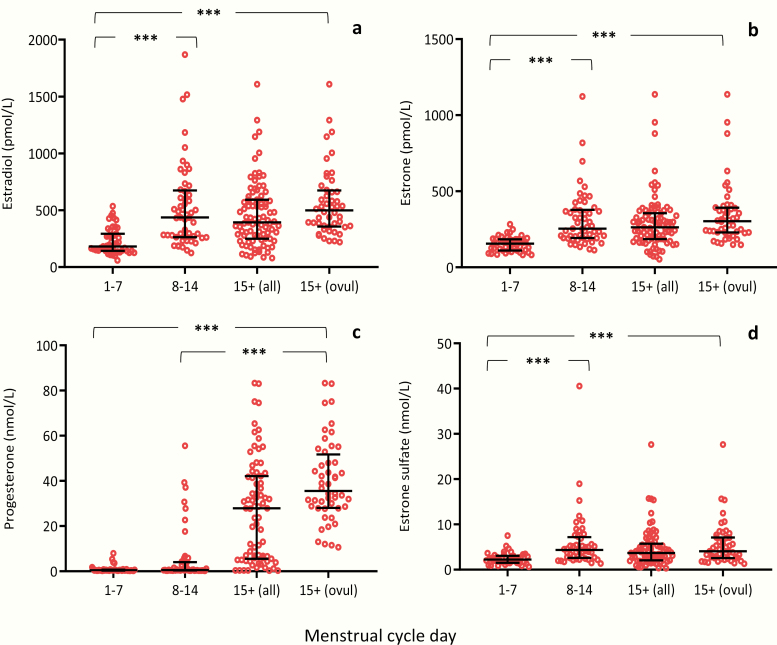
Female serum concentrations of (a) estradiol (E2), (b) estrone (E1), (c) progesterone, and (d) estrone sulfate (E1-S) as a function of menstrual cycle days in 188 women aged 24.7 to 43.9 years; early follicular phase from days 1 to 7, n = 46 (a,b) and n = 41 (c,d); late follicular phase from days 8 to 14, n = 53 (a,b) and n = 48 (c,d); luteal phase post ovulation from day 15 (15+ all), n = 89 (a,b) and n = 75 (c,d); and from day 15, at which ovulation was confirmed by a progesterone concentration >10 nmol/L (15+ ovul.), n = 48 (a,b). Bars represent median, 25, and 75 percentiles. ***P < 0.001.

Serum concentrations of E1, E2, progesterone and E1-S in the early follicular phase (days 1–7), late follicular phase (days 8–14), and luteal phase (day 15+) are shown in Fig. 7 and (26), while serum concentrations of free E1, free E2, 17-OH-P, androstenedione, testosterone, and DHEAS are shown in (26). In general, all steroid hormones increased significantly from the early follicular phase (days 1–7) to the late luteal follicular phase and further on to the luteal phase, during which the post-ovulatory state was confirmed by progesterone concentrations >10 nmol/L. Furthermore, significantly higher levels were observed for all steroid hormones (except for progesterone and DHEAS) in the late follicular versus the early follicular phase. Progesterone and 17-OHP were significantly higher in the luteal phase than in the late follicular phase.

### Association to oral contraceptives

**Figure 8.**
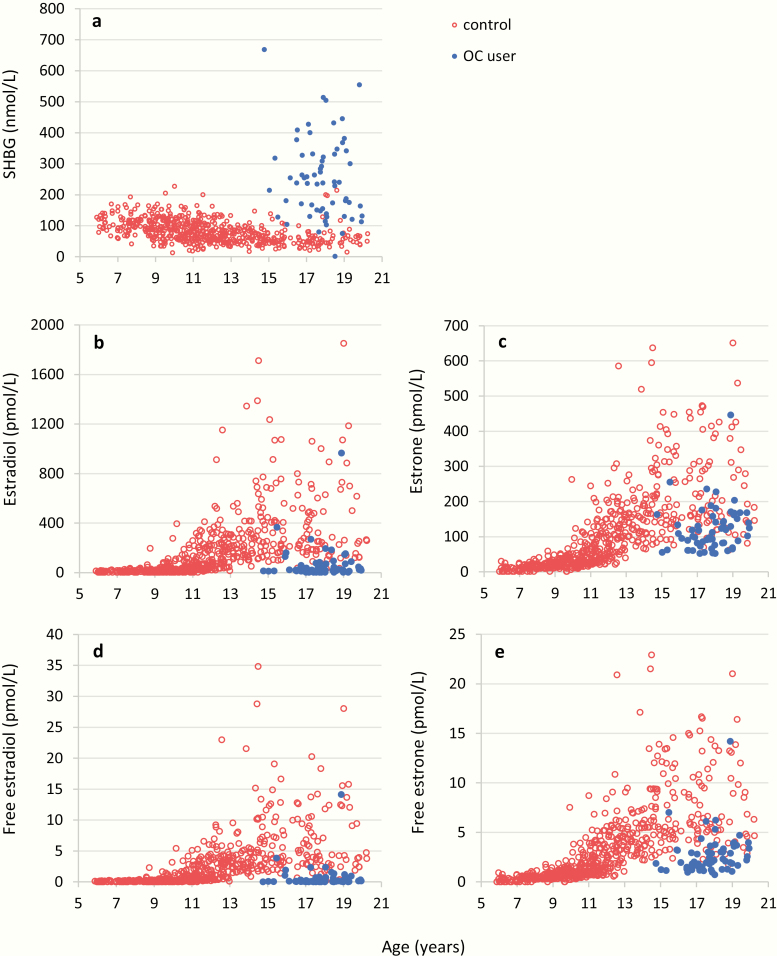
Female serum concentrations (n = 666) of (a) sex hormone binding globulin (SHBG), (b) estradiol (E2), (c) estrone (E1), (d) free estradiol (free E2), and (e) free estrone (free E1) by age, separated into users (blue dots, n = 62) and nonusers (red dots, n = 603) of oral contraceptives (OC).

Use of oral contraceptives was significantly associated with increased SHBG concentration of, very low total and free E2 concentrations as well as lower total and free E1 than nonusers (Fig. 8).

## Discussion

In this study, we present detailed sex-, age-, and puberty-stratified reference ranges for serum concentrations of E1, E2, free E1, and free E2 from the age of 3 months to 60 years. E1 and E2 concentrations were determined by state-of-the-art LC-MS/MS methodology.

### Infancy

In infants, the pituitary–gonadal hormone axis is activated for a brief period of time often referred to as “mini-puberty.” In accordance, we observed on average 2.5 times higher E2 concentrations in infant girls than in older prepubertal girls. This finding is in line with previous observations using immunoassays (34). These higher E2 concentrations are seen concomitantly with elevated FSH and LH concentrations (35) and could in part be responsible for the transient thelarche observed in many infant girls. In contrast to infant girls, E2 was detectable in only a few infant boys ranging from <LOD to a maximal of 10 pmol/L. Thus, we found a clear sex difference in E2 concentrations in infants, a finding in contrast to another recent LC-MS/MS-based study by Bae et al., which included infants at the same age as in our study (36). Bae et al., reported comparable E2 concentrations in infant boys and girls ranging from LOD (37 pmol/L) to about 80 pmol/L. Furthermore, they showed that several boys from 1 to 5 years had detectable E2 concentrations in the range of 37 to 71 pmol/L, whereas most girls at the same age had almost no detectable E2 concentrations (36). To our knowledge the study by Bae et al. (36) is the only one to show high E2 concentrations in infants of both sexes and younger boys. We have no explanation for this disagreement other than the considerable difference in LOD values between studies.

### Childhood

We found detectable E2 concentrations in more than half of the prepubertal girls aged 1 to 7 years, but E2 concentrations did not exceed 20 pmol/L. Reference ranges have been reported in smaller cohorts of healthy children using immunoassays with varying detection limits, for example, <36.7 pmol/L (37), <6 pmol/L (38,39), and <3.7 pmol/L, when purification steps were performed before the measurements (40). With these immunoassays, no significant differences in E2 concentrations between prepubertal boys and girls were observed. In contrast, using our specific and highly sensitive LC-MS/MS method, markedly higher (and detectable) concentrations were measured in prepubertal girls than in prepubertal boys in whom most values were below the detection limit. Thus, even before any physical signs of pubertal maturation were present, girls had significantly higher E2 concentrations than boys. In 1994, Klein and co-workers (41) developed an ultrasensitive recombinant cell bioassay for assessment of estrogenic activity with a detection limit for E2 in the range of 0.07 to 0.7 pmol/L. In accordance with our present study, they reported significantly higher estrogen bioactivity in prepubertal girls (approximating 5.6 pmol/L) than in prepubertal boys (approximating 1.5 pmol/L) (42). Subsequently, Paris and co-workers (43) reported average estrogenic activity in sera from prepubertal girls approximating 13.0 pmol/L. We have previously measured prepubertal E2 concentrations using a GC-MS/MS methodology with a detection limit of 1.8 pmol/L (44), and the results shows a significant prepubertal difference by sex. We speculate that the higher E2 concentrations in prepubertal girls than in boys, although low, are likely to play a biological role and might contribute to the earlier-onset sexual maturation in girls compared with boys. This sex difference in low prepubertal E2 concentrations may be obscured by cross-reactivity to other steroids in the less specific immunoassays. This underlines the importance of adequate E2 reference values when evaluating children with precocious puberty. Accordingly, pediatric reference ranges for E2 using appropriate LC-MS/MS methodology have been requested from the Endocrine Society (1–3), which was also the motivation for our present study. In clinical practice, premature breast development is a frequent reason for referral. The availability of a highly sensitive and specific method could provide a tool for better differentiation of premature thelarche and slowly and rapidly progressing precocious puberty.

### Puberty

As expected, E2 concentrations increased with higher breast stage in pubertal girls. However, in accordance with our previous findings for urinary LH and FSH excretions, E2 concentrations rose significantly with increasing age, even before physical signs of puberty were evident (45). The large interindividual variation in the circulating total E2 levels in girls within the same pubertal stage may reflect varying estrogen receptor sensitivity or differences in circulating free E2 concentrations influenced not only by total E2 concentrations, but also by SHBG, albumin and E2 clearance. Our breast stage–specific reference ranges might be useful when inducing pubertal development in hypogonadal girls during which attainment of physiological circulating concentrations of E2 according to age and pubertal stage is wanted.

### Menstrual cycle and oral contraceptive use

We describe reference ranges according to menstrual cycle day for concentrations of E1, E2, and E1-S determined by LC-MS/MS, and we found low levels of E1, E2, and E1-S in the early follicular phase, which increased in the late follicular phase as expected. Significantly higher E1, E2, and E1-S levels were maintained in the luteal phase. This pattern is in accordance with previous reports using immunoassays (46); however, absolute concentrations may differ. Importantly, our present results are in accordance with reference ranges from the only existing paper on E2 determined by LC-MS/MS across the menstrual cycle (47). This study exhibits the limitation that we did not collect daily blood samples throughout the menstrual cycle but had to group subjects into early/late proliferative phase and luteal phase, as we only had one blood sample for each woman. Use of oral contraceptives in healthy teenage girls markedly suppressed serum concentrations of total and free E2, as the synthetic ethinyl-estradiol from oral contraceptive does not cross-react in our LC-MS/MS methodology. Lower total and free E2 in oral contraceptive users are also seen in women with polycystic ovary syndrome in whom oral contraceptive are used to reduce free androgens by increasing SHBG levels and inhibiting ovarian steroid synthesis. In order to interpret results correctly, it is therefore important to know if a woman is using oral contraceptive or not to interpret results correctly.

### Postmenopausal women

We found detectable E2 concentrations in 94% of the 55- to 61-year-old women ranging between 7 and 80 pmol/L, which is in accordance with Thurston et al. (48). Interestingly, E2 levels were significantly lower than E1 concentrations. In postmenopausal women, DHEA and its derivative androstenedione are considered major sources of circulating E1 from aromatization in extragonadal tissues, whereas residual E2 secretion primarily originates from the ovaries. This finding is in accordance with recent data from an Australian study, which reported increasing E1 concentrations determined by LC-MS/MS with increasing age in 70 to 85+ years old women (49). In the present study, a few of the postmenopausal women had very high E1 levels, for which we have no obvious explanation, but unreported intake of oral estrogens cannot be ruled out.

### Men

Assessment of circulating E2 concentrations in men could be useful in various clinical situations including gynecomastia, hypogonadism, and osteoporosis. Elevated concentrations are observed in patients with gynecomastia due to human chorionic gonadotropin (hCG)-producing testicular tumors, but the diagnostic value of E2 quantification by LC-MS/MS in men with idiopathic gynecomastia remains unsolved. Likewise, E2 concentrations are reported to be low in hypogonadal boys and men and are—at least in part—associated with low bone mineral density (50). A genome-wide association study (GWAS) of more than 11 000 European men supports the causal importance of E2 for bone health in men (51). However, exploration of the role of variation within the low male levels of E2 has partly been hampered by the insensitivity of most conventional immunoassays. So far, the existence of valid reference ranges for circulating E1 and E2 concentrations in males using LC-MS/MS are sparse. In the current study, circulating E2 concentrations ranged from 50 to 150 pmol/L, with no changes across adult ages. E1 concentrations did not significantly vary with age in 30- to 60-year-old men but were higher than their E2 concentrations. The concentration levels for both E1 and E2 is in accordance with median concentrations observed in a previous study by Jasuja et al. (52), who also used LC-MS technology. However, in contrast to our study, Jasuja and co-workers observed slightly increasing median concentrations for both E1 and E2 in men aged 30 to 80+ years (52). The clinical and diagnostic role of circulating E1 in men remains to be explored.

### Methodological aspects

By using isotope-diluted online TurboFlow-LC-MS/MS with prior liquid–liquid extraction, we were able to develop a very sensitive and robust method. The present estrogen method attained all the requirements for modern high-throughput methods: high accuracy, precision, and sensitivity with low LOD and LOQ. We avoided the time-consuming derivatization process (14,53). Instead, the sample preparation was improved by an ordinary liquid–liquid excretion step, and the sensitivity was increased by using an ammonium fluoride buffer together with a weak basic methanol buffer as mobile phases on the eluting column. Furthermore, the ionization was improved by using a HESI probe operating in negative mode.

Several new LC-MS/MS-based methods for multiple detections of both androgenic and estrogenic steroids have recently been developed (4,14,54). However, simultaneous measurement of multiple steroids often comes at the cost of decreased assay sensitivity. Our main goal was to develop an ultrasensitive method for E2 with detection limits in the low picomolar range suitable for clinical pediatric endocrinology, while still having the range to cover E2 levels throughout the whole lifespan in both sexes. When considering the relatively low serum volume needed in our estrogen method, the LODs were similar to or even lower than many other recently published methods also using LC-MS/MS technologies with presenting liquid–liquid extraction (4,11,15). We would have preferred also to include E1S in this method. However, it was not possible for us after the specific liquid–liquid extraction step to reach an acceptable E1S signal compared with the E1-S signal in our already well-established method for androgens, corticosteroids, and E1-S (55) without compromising the sensitivity for E2.

We decided to calculate free E1 and E2 using the equation of Mazer (31) and to provide reference ranges throughout life in both sexes. To our knowledge, such reference ranges have not previously been reported. The free E1 and E2 concentrations mostly mimic total hormone concentrations. We speculate that they will, together with gonadotropin measurements, provide a future base for individualized hormonal treatment regimens. However, their clinical value remains to be evaluated in various clinical situations.

In conclusion, we present a novel highly sensitive LC-MS/MS for simultaneous quantification of E1 and E2 (and E3) and present reference ranges throughout life with highly significant sex dimorphic changes with age, pubertal stage, menstrual cycle, and menopausal status.

## Abbreviations

17-OHP: 17-hydroxyprogesterone
CV: coefficient of variation
DHEAS: dehydroepiandrosterone sulfate
E1: estrone
E1-S: estrone sulfate
E2: estradiol
E3: estriol
FSH: follicle-stimulating hormone
GC: gas chromatography
HESI: heated electrospray ionization source
LC-MS/MS: liquid chromatography tandem mass spectrometry
LH: luteinizing hormone
LOD: limits of detection
RIA: radioimmunoassay
SHBG: sex hormone-binding globulin
T: testosterone.

## Additional Information

***Disclosure Summary:*** The authors have nothing to disclose.

***Data Availability:*** All data generated or analyzed during this study are included in this published article or in the data repositories listed in References.
